# Hydrogel Formulation for Biomimetic Fibroblast Cell Culture: Exploring Effects of External Stresses and Cellular Responses

**DOI:** 10.3390/ijms25115600

**Published:** 2024-05-21

**Authors:** Immacolata Greco, Hatim Machrafi, Christophe Minetti, Chiara Risaliti, Allegra Bandini, Francesca Cialdai, Monica Monici, Carlo S. Iorio

**Affiliations:** 1 Center for Research and Engineering in Space Technologies, Universit libre de Bruxelles, 1050 Brussels, Belgium; immacolata.greco@ulb.be (I.G.); christophe.minetti@ulb.be (C.M.); allegra.bandini@stud.unifi.it (A.B.); carlo.iorio@ulb.be (C.S.I.); 2 GIGA-In Silico Medicine, University of Liège, 4000 Liège, Belgium; 3 ASAcampus Joint Laboratory, ASA Research Division, Department of Experimental and Clinical Biomedical Sciences “Mario Serio”, University of Florence, 50139 Florence, Italy; chiara.risaliti@unifi.it (C.R.); francesca.cialdai@unifi.it (F.C.); monica.monici@unifi.it (M.M.)

**Keywords:** hydrogels, bioscaffolds, tissue engineering, fibroblast, parabolic flight, mechanical stress, gravity-related stress

## Abstract

In the process of tissue engineering, several types of stresses can influence the outcome of tissue regeneration. This outcome can be understood by designing hydrogels that mimic this process and studying how such hydrogel scaffolds and cells behave under a set of stresses. Here, a hydrogel formulation is proposed to create biomimetic scaffolds suitable for fibroblast cell culture. Subsequently, we examine the impact of external stresses on fibroblast cells cultured on both solid and porous hydrogels. These stresses included mechanical tension and altered-gravity conditions experienced during the 83rd parabolic flight campaign conducted by the European Space Agency. This study shows distinct cellular responses characterized by cell aggregation and redistribution in regions of intensified stress concentration. This paper presents a new biomimetic hydrogel that fulfills tissue-engineering requirements in terms of biocompatibility and mechanical stability. Moreover, it contributes to our comprehension of cellular biomechanics under diverse gravitational conditions, shedding light on the dynamic cellular adaptations versus varying stress environments.

## 1. Introduction

Tissue engineering is at the forefront of biomedical research and presents promising answers to major problems in personalized healthcare and regenerative medicine [1,2]. The multidisciplinary area of tissue engineering combines concepts in biology, engineering, and material science to create biological replacements that can preserve, enhance, or repair tissue function. Creating functioning organs and tissues that may be inserted into the body to replace diseased or damaged tissue or to promote tissue regeneration is the ultimate goal of tissue engineering [3]. One of the main components of an engineered tissue is a scaffold, which serves as the structural framework for tissue regeneration. Central to this field is the development of scaffolds capable of mimicking the complex microenvironment of native tissues, fostering cellular interactions, and guiding tissue regeneration [3,4].

The polymeric scaffold is an essential component in nearly all tissue engineering strategies, serving multifaceted roles akin to the extracellular matrices in tissues. Extracellular matrices establish connections between cells, regulate tissue architecture, modulate cellular activities, and facilitate the transport of vital nutrients, metabolites, and growth factors [5]. Diverse amino acids and sugar-based macromolecules in these matrices play a necessary role in adequately functioning and growing new tissue in tissue engineering hydrogels. These hydrogels must adhere to stringent design criteria encompassing both traditional physical attributes, such as durability and mechanical properties, and biological performance parameters, like cell adhesion [6].

Among the materials extensively explored within tissue engineering, hydrogels occupy a prominent position due to their polymer network’s capacity to absorb substantial quantities of fluids [7,8,9]. The pivotal factor in evaluating the utility of hydrogels lies in their biocompatibility, signifying their ability to function as intended within the body without instigating significant scarring, harm to neighboring cells, or any other adverse reactions that might impede their intended purpose [10]. While many polymer types have been studied and employed in tissue engineering endeavors, not all meet mechanical robustness and biocompatibility requirements [6]. Tissue engineering is a field that aims to create functional replacement tissues or organs for medical purposes. This involves the use of biomaterials, cells, and growth factors to regenerate or repair damaged tissues [11,12]. 

Preparation methods for synthetic hydrogels are multiple but can, in most cases, be summarized by two types, i.e., through chemical and physical cross-linking techniques. Hydrogels that are chemically cross-linked are often produced by chain-to-growth polymerization, of which UV polymerization is a widely used technique [13]. Physical cross-linking of hydrogels can occur through non-covalent H-bonding or ionic interactions, the latter of which is of interest in this work. The benefit of synthetic polymers is their capacity to be tailored to certain physical features, like mechanical characteristics, to fit different applications. However, some complications to their use can be mentioned, such as toxic degradation products and the loss of mechanical qualities brought on by deterioration [14]. Another possible drawback of the use of synthetic hydrogels in scaffolds for tissue engineering is limited biocompatibility, in contrast to many natural hydrogels [15]. One of the most used synthetic hydrogels is PEGDA (poly(ethylene glycol) diacrylate), as they possess the necessary mechanical characteristics [16]. However, they do not present high biocompatibility. This latter characteristic is amply fulfilled by natural polymers, such as sodium alginate (SA), which is often used for its easy gelation and strong biocompatibility. However, they do not possess the necessary mechanical properties for biological tissues that are of interest in tissue regeneration [17]; instead, they display viscoelasticity, nonlinearity, and mechanical anisotropy [18]. In this work, it is proposed to combine the mechanical characteristics of PEGDA with the remarkable biocompatibility of SA in a double network (DN), to obtain the properties required for materials intended for tissue engineering [19]. Two different polymer networks make up a DN hydrogel: one is brittle and stiff, while the other is soft and ductile. The DN hydrogel’s entanglement of these cross-linked networks provides a balanced combination of high strength and biocompatibility [14].

One of the principal needs in cell survival is the presence and transport of proteins. This means that the hydrogel scaffold needs to include other components. The first is Gelatin, which is a protein derived from collagen, while the second is poly-L-lysine, a synthetic polymer composed of the amino acid lysine. Both gelatin and poly-L-lysine are fundamental materials for the cells’ adhesion and proliferation [20,21]. The cells used in this work are fibroblasts, which are a type of cell that plays a crucial role in the formation and maintenance of connective tissues in the body. Indeed, in tissue engineering, fibroblasts are mostly used as a cell source to populate and remodel the hydrogel scaffold. They are an important cell source for the regeneration of connective tissues, while hydrogels offer a flexible substrate for tissue engineering. When coupled, they present a viable method for developing useful, bio-inspired tissues for future medical use [22]. The double-network approach has already been studied [23]. However, the presented solutions did not include cell ligands (such as gelatin) to promote cell adhesion. The novelty of the presented work is to fulfill the requirements of tissue engineering applications by using a double network hydrogel including compounds that promote cell adhesion. Sodium alginate, PEGDA, and gelatin will form the polymeric hydrogel scaffold under study in this work, and the preparation method will be explained later in the text. 

Fibroblast cells exhibit extremely dynamic and tightly regulated behavior under stress during tissue healing, which enables them to react adaptively to many environmental stimuli, such as mechanical strain and tissue injury. In practical tissue engineering applications, various stresses can arise (such as mechanical [24,25] and altered-gravity [26] stresses) during the utilization of biomimetic scaffolds, which potentially affect cell viability within the scaffold. More specifically, mechanical stresses cause a reduction in cell growth and an increase in cell senescence, which lowers the viability of the cells. Therefore, we are interested in studying the responses of fibroblast cells in a tissue-like environment provided for by the hydrogel scaffold under such external stresses. Understanding how these stresses affect the behavior of the cell–scaffold system is fundamental for optimizing tissue-engineering strategies and ensuring successful tissue regeneration.

A particular type of stress that is of interest here, besides the mechanical one, is the type exerted by altered gravity on tissue-engineered constructs. Given its impact on cellular behavior, morphogenesis, and tissue development, gravity does, in fact, play a critical role in tissue engineering. The altered-gravity-induced stresses can be studied by means of parabolic flights. A parabolic flight campaign enables the execution of experiments under both hypergravity (approximately 1.8–2 times Earth’s gravity, denoted as g_0_) and microgravity (approximately 0.001 g_0_) conditions. The campaign involves 31 parabolic flight maneuvers, during which hypergravity is experienced for approximately 20 s and microgravity for around 22 s for each parabola [27]. Microgravity and altered gravity can have a significant influence on different biological systems. This means that in order to study the behavior of a cell–scaffold system, which could lead to proposing ideal scaffolds, such a study should be done under realistic conditions, i.e., under altered-gravity and microgravity conditions [7,28]. For this reason, the purpose of this study is to investigate the behavior of a cell–scaffold system monitored by adherent fibroblast cells’ behavior on biomimetic hydrogels under stress. For the hydrogel, a novel biomimetic material that fits the requirements for tissue engineering application is used. Both solid and porous hydrogel architectures were used in the fabrication of the hydrogels. The solid hydrogels allow a better visualization of the cell behavior, while the porous ones mimic better the material used for tissue engineering. Stressors include mechanical stress applied through tensile elongation and changing gravity caused by the parabolas. After the end of each parabolic flight day, a “cell Imaging Kit” in conjunction with “Nikon microscope’s Epi Fluorescence attachment” have both been used to analyze the fibroblast response to the altered gravity. This has been done by visualizing the morphology of the fibroblast cells seeded on the hydrogel samples. 

## 2. Results and Discussion

The response of cells to gravity varies among different cell types, especially when they are exposed to altered-gravity conditions [29]. We consider here two types of stresses to which the cells can respond, i.e., mechanical stress by stretching and stress caused by a gravity level that undergoes changes from microgravity to double the normal gravity in several subsequent cycles. We present the results using these parameters for both solid hydrogel and porous hydrogel configurations. 

### 2.1. Biocompatibility

**Figure 1:**
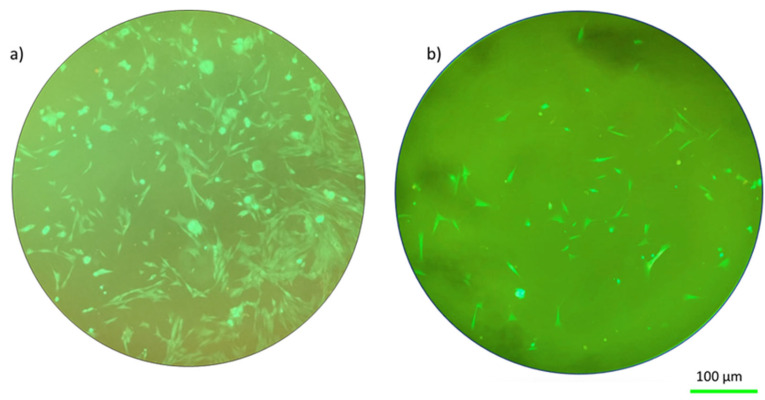
Fluorescence images of living cells on hydrogels in relaxed conditions: (**a**) solid hydrogel on the ground, (**b**) porous hydrogel on the ground.

The biocompatibility of a hydrogel scaffold is fundamental for tissue engineering applications. Before the hydrogels were sent for further investigations, it had been verified that the hydrogel used in this work was biocompatible [30]. The fluorescent dyes were used to investigate the cell viability of the hydrogel after 48 h of incubation. Additionally, a visual examination of the morphology of the cells can reveal information about how well fibroblasts were seeded. In particular, spindle-shaped morphology and strong substrate adherence are characteristic of healthy fibroblasts [22]. The image manifested that the developed double network hydrogel was biocompatible to cells. In addition, the material’s biocompatibility is demonstrated by the cellular shape seen in Figure 1 and the uniform green fluorescence that is characteristic of living cells.

### 2.2. Relaxed Conditions at Normal Gravity

Typically, fibroblast cells in optimal conditions exhibit an elongated morphology (as shown in Figure 1. Figure 1 shows these cells under relaxed conditions for both the solid hydrogel and porous hydrogel configurations.

### 2.3. Effect of Mechanical Stress at Normal Gravity

**Figure 2:**
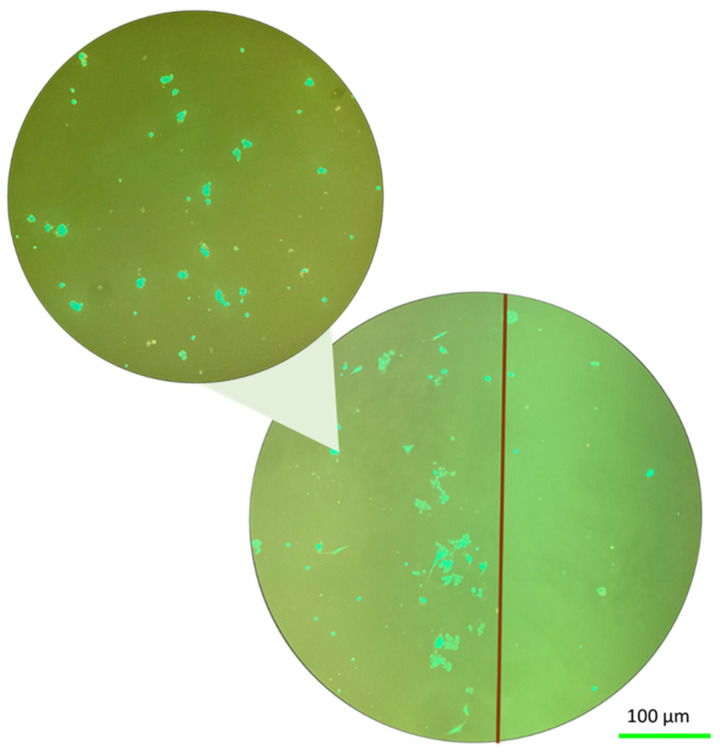
Fluorescence images of cells on the solid hydrogels after applied mechanical stress at normal gravity. It shows aggregation at the sample’s edge. A zoom of the fluorescence image shows that the cells form multicellular aggregates.

**Figure 3:**
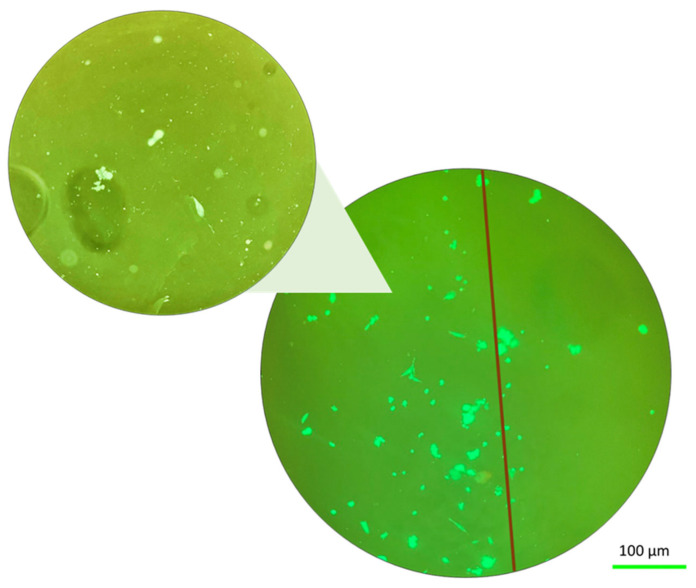
Fluorescence images of cells on the porous hydrogels after applied mechanical stress at normal gravity. It shows aggregates at the sample’s edge. A zoom of the fluorescence image, in a different focus level, shows that the cells form multicellular aggregates.

Having established the adhesion of Fibroblast cells on the material, we concentrated on the effect of two types of stresses. In this section, we present microscope images of fibroblast cells on hydrogels after applying mechanical stress. The morphological appearance of the cell-hydrogel system is shown in Figure 2 for the solid hydrogel configuration. By examining the cell distribution of the fibroblast cells that are seeded on top of the solid hydrogel, as indicated in Figure 2, it is possible to notice that the tensile stress induces an effect on the seeded cells. In these stress conditions, it was observed that these cells respond to the elasticity of the sample by detaching from the sample and forming 3D agglomerates. In this test, the fibroblast cells exhibited a rounded and aggregated morphology, deviating from their usual elongated form. In addition, the cells, because of the tensile stress, tend to go to the edge of the sample where the stress line is typically higher [31,32]. In the margin of the sample, demarcated by the red line in Figure 2, there is a higher concentration of cells mainly situated along the hydrogel edge [25]. Figure 2 provides a 10× magnified view of the fibroblast cells after the test, showing the presence of aggregates. As shown in Figure 3, for the porous hydrogel sample, we experienced the same cell behavior as for the solid hydrogel sample. The seeded cells exhibit a propensity to aggregate, with a notable tendency to redistribute towards the edge of the sample. A key distinction between the solid hydrogel and porous hydrogel samples, however, lies in the interconnected pores present in the porous hydrogel structure. The porous hydrogel exhibits a sponge-like structure characteristic of scaffold-like hydrogels. Consequently, the cells are distributed across multiple layers, and not all of them are observable within a single focal plane. 

### 2.4. Effect of Mechanical Stress with Altered-Gravity-Related Stress

**Figure 4:**
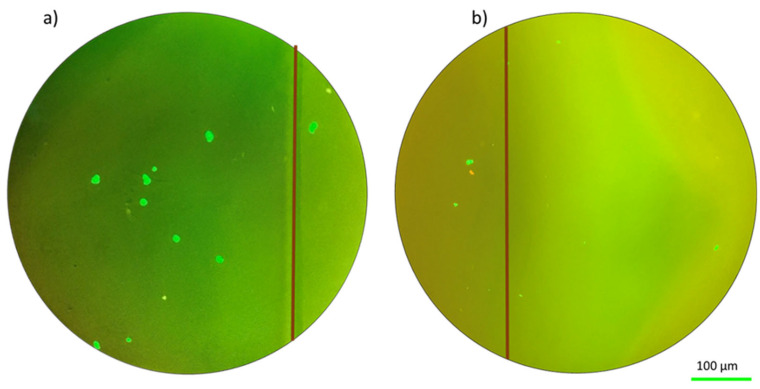
Fluorescence images of cells at altered gravity for the relaxed case (without any applied tensile stress) for both the solid hydrogel sample (**a**) and the porous hydrogel sample (**b**).

Here, we are interested in the effect that mechanical stress has on the fibroblast cells behavior while they are also under gravity-related stress. Figure 4 shows the results for the behavior of the fibroblast cells under gravity-related stress but with no mechanical stress (the “relaxed” sample).

**Figure 5:**
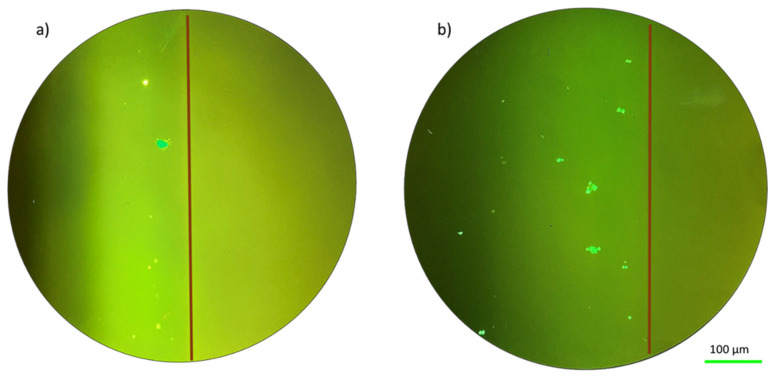
Fluorescence images of cells at altered gravity for the stressed case (with applied tensile stress) for both the solid hydrogel sample (**a**) and the porous hydrogel sample (**b**).

The fluorescence images in Figure 4 of the solid hydrogel and porous hydrogels were taken after the parabolic flight (as the images could not be taken during the flight). It can be seen that some of the fibroblast cells redistributed towards the edge of the sample in both the solid and porous hydrogel configurations. The effect is not large but still significant. The reason for the small number of cells visible in the images will be discussed later. Figure 5 shows the same results but applying mechanical stress at the same time. These images emphasized the ability of cells to adapt to environmental challenges by changing their morphology and forming aggregates [33]. 

In the case of Figure 5, there is a combination of two different stresses: altered-gravity-related stress due to the parabolic flight and tensile stress due to the elongation applied during the flight. Figure 5 shows a redistribution of the fibroblast cells in particular in the proximity of the edge of the sample. From the presented results, it is possible to highlight the impact of parabolic flight conditions and mechanical stress on fibroblast cell morphology and distribution. In addition, the results emphasize the ability of cells to adapt to environmental challenges by changing their morphology and forming aggregates. Under the two types of investigated stresses, the fibroblast cells redistribute towards the edge of the hydrogel samples. Table 1 shows the summary of the experienced results. Some additional elements observed in the results will be discussed in the next section.

### 2.5. Post-Stress Conditions 

**Figure 6:**
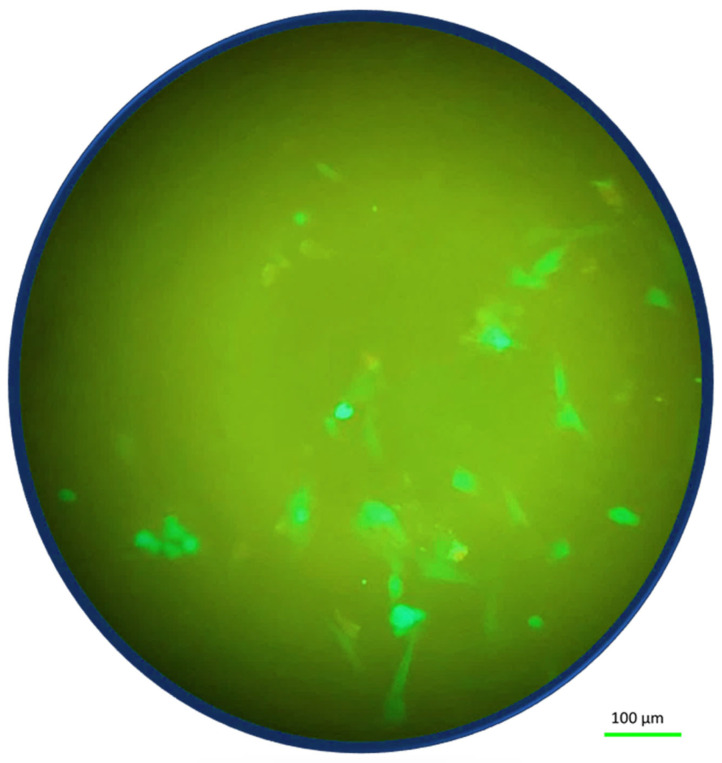
Fluorescence image of a solid hydrogel sample 6 h after removing altered-gravity and mechanical stresses.

In the framework of the project, the images were taken immediately after the experiments to demonstrate the effect of the altered-gravity and mechanical stresses on the cells. However, in the hours that follow the end of the experiments (in the absence of any stress), it is interesting to see whether the cells die or are able to return to normal morphology. An example is shown for the solid hydrogel sample, where a fluorescence image is taken 6 h after the end of the experiments in Figure 6. The fluorescence image shows that after 6 h, the morphology of the cells started to be elongated again, suggesting a return to normal morphology after the absence of the stresses.

### 2.6. Mechanical Properties

**Figure 7:**
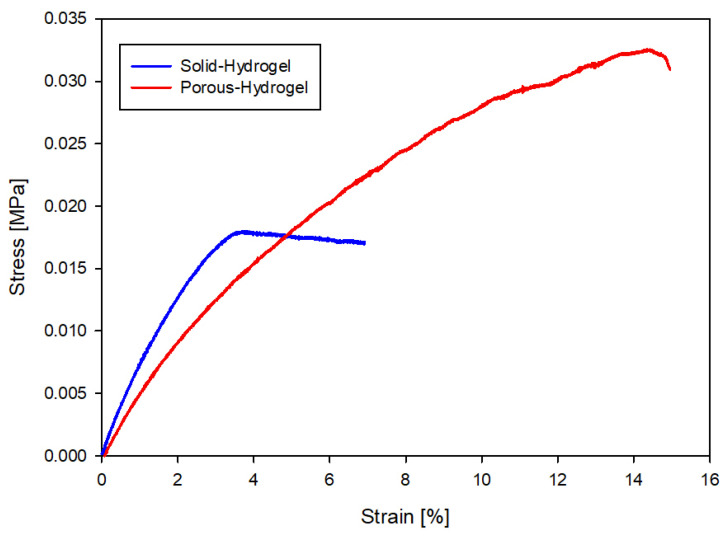
Stress–strain curves of the solid and porous hydrogel samples.

The mechanical properties of hydrogels play a crucial role in governing the interactions between cells and the extracellular matrix. While the objective of tissue engineering is to replicate organ behavior, it is essential to optimize the mechanical properties of the tissue. This should not only be based on human physiology but also on the practicality of surgical manipulation of such grafts within the human body. Therefore, achieving higher elasticity and improved mechanical properties becomes imperative. Within this framework of wound healing, Young’s modulus can vary from 0.2 to 1 MPa [34]. During a previous parabolic flight campaign, the mechanical properties of PEGDA hydrogels have been tested. It appeared that altered gravity did not influence the mechanical properties of the hydrogels. This justifies measuring the elasticity of the hydrogel in this work on the ground. Figure 7 shows the corresponding stress–strain curve of the solid hydrogel and porous hydrogel samples, leading to Young’s moduli of, respectively, 0.55 MPa and 0.48 MPa. The values presented for this study align closely with the requirements of wound-tissue functionality and medical standards [35]. 

### 2.7. Morphology

**Figure 8:**
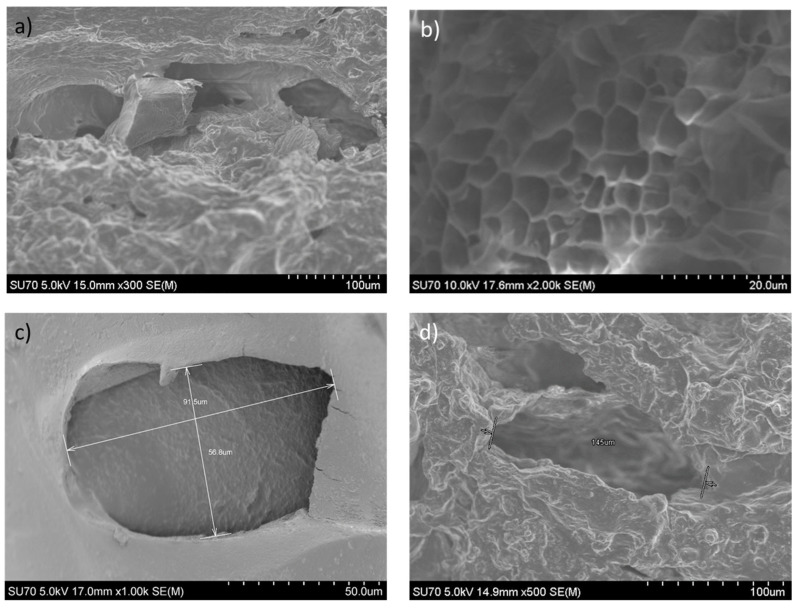
SEM images of a porous hydrogel sample: (**a**) example showing the interconnectivity of the pores, (**b**) sample section, representative of a sponge-like structure, typical for scaffold-based materials; (**c**) a zoom on an isolated pore with maximum pore size of around 92 µm; (**d**) a typical pore shape with maximum pore size of 145 µm.

Figure 8 shows an SEM image of the porous hydrogel. Up to three pores were measured throughout the whole sample, and their average size was 120 µm, which fits the requirements for wound healing tissue engineering [36]. Figure 8a,c show the inside pores of the hydrogel’s structure, where Figure 8a focuses on the interconnectivity between several internal (smaller) pores and Figure 8c zooms into one other isolated pore. The interconnectivity is especially assured by the smaller pores, making part of a sponge-like structure, as shown in Figure 8b, typical for a scaffold-based material [37]. However, the average value of the pore size is actually larger. Indeed, Figure 8c,d are representations of the pore shapes and sizes, 92 µm and 145 µm, respectively, recalling the mean value of about 120 µm. 

### 2.8. Points of Discussion

This work stems from the research theme of the WHISKIES (Wound Healing And Monitoring In Space) project funded by ESA (European Space Agency). As part of the project, this study’s main goal was to create a biomimetic hydrogel that supports the growth of fibroblast cells. Our specific objectives were to assess its mechanical characteristics in the context of skin stretching simulation and to examine the behavior of cells in conditions of changed gravity. 

In this study, we developed a hydrogel-based material tailored to fulfill the requirements of mechanical and biocompatible properties for tissue engineering applications. In addition to being used as a scaffold for regenerative medicine, it is fundamental that the biomimetic material shows biocompatibility. Fluorescence images were taken to test the cells’ morphologies after applying mechanical and altered-gravity condition stresses.

When examining the fluorescence images of the fibroblasts that were grown on the solid and porous hydrogels, some additional particularities are to be discussed. Depending on the imposed stress conditions, different behaviors are displayed by the cellular morphology. Under normal circumstances, fibroblasts cultivated on solid and porous hydrogels showed a homogeneous distribution over the hydrogel surface and inside, respectively. Instead of the usual single-celled structure seen under control conditions, cell clustering occurred under tensile stress conditions, which resulted in the creation of cellular aggregates. This phenomenon was more prominent under altered-gravity stresses. Under these conditions, cells tended to redistribute towards the edge of the sample. 

The cell viability study can be improved by investigating metabolic characteristics. However, within the framework of the platform available for the experiments, only a qualitative representation of the cell’s viability was possible. In this light, the images that have been shown in this work were analyzed by the Imaging Kit to count the number of live and dead cells for the determination of the percentage of live cells. The percentage of cell viability can be calculated as [100 × (total cells minus dead cells)/total cells] [38]. For the stressed sample tested in normal gravity, the viability of the cells reached (80 ± 5)%, while for the samples tested in altered-gravity conditions, because of the temperature’s fluctuation, the viability reached (49 ± 5)%. Indeed, the airplane’s inside temperature was 7 °C on the morning of the flight campaign. It took some time for the temperature to rise to 37 °C because of the plane’s initial low temperature and the hydrogels’ reactivity to it. The reduced cell count seen in settings of changed gravity may have resulted from this temperature fluctuation’s effect on the survivability of the cells. Tests at optimal temperatures have been performed, where no significant supplementary cell death was observed whether the samples were under stress or without stress. This suggests that a prolonged lower-temperature environment was the main cause of cell death in this work. Even though the low temperature induced the cells to suffer, it was still observed that the cells tended to form aggregate. Notable morphological changes were seen after being exposed to both stressors, which include mechanical stress from tensile forces and parabolic flight-induced gravity stresses. The morphology of fibroblasts was rounded, and the cells formed aggregates, which were different from their typical elongated appearance. In addition, cell migration was observed towards the side of the sample where the stresses were stronger.

The results presented in this paper highlighted the impact of parabolic flight conditions and mechanical stress on fibroblast cell morphology and distribution [26]. It was also demonstrated that under the two types of investigated stresses, the fibroblast cells redistribute towards the edge of the hydrogel samples.

## 3. Methods and Materials

### 3.1. Materials

#### 3.1.1. Hydrogel Materials

Sodium alginate (SA), Poly(ethylene glycol)-diacrylate (PEDGA) (average Mn700), and Igracure 2959 (2-Hydroxy-4’-(2-hydroxyethoxy)-2-methylpropiophenone) (I2959), were purchased from Sigma-Aldrich (Belgium). Calcium chloride dehydrate (CaCl_2_∙2H_2_O), Gelatine (Gel), and Poly-L-lysine were purchased from VWR (Belgium). The CaCl_2_ solution was prepared by dissolving CaCl_2_ powder into distilled water to obtain a final concentration of 2.5% (*w*/*v*). 

#### 3.1.2. Cells Culture Materials

The cell line used is Normal Human Dermal Fibroblasts (nHDF) from ATCC (USA), while Dulbecco’s modified Eagle’s medium (DMEM) cell medium, Bovine Fetal Serum (FBS), 200 mM aqueous solution of L-glutamine, phosphate-buffered saline solution (PBS) with pH 7.4, Tripsin 0.05%-EDTA 0.02% water solution and 10 mg/mL aqueous solution of Penicillin–Streptomycin was purchased from Sigma-Aldrich. The final cell medium used for the cells’ culture was made by adding 100 U/mL of, respectively, penicillin and streptomycin in the cells’ medium (DMEM) to avoid any bacteria contamination. 

#### 3.1.3. Hydrogels Preparation

PEGDA was first dissolved in distilled water at a 1% (*w*/*v*) concentration, and the photo initiator I2959 was added to the PEGDA precursor solution at a 0.6% (*w*/*v*) concentration. After that, a double network solution was obtained by subsequently adding 4% (*w*/*v*) of SA, 4% (*w*/*v*) of Gel, and 0.1% (*w*/*v*) of poly-L-lysine (acting as a protein contribution) to the hydrogel solution. All the solutions, before cross-linking, were stirred at 40 °C to dissolve the gelatine until they became homogeneous and degassed for 2 h to eliminate air bubbles. The solutions were then polymerized by ultraviolet (UV) irradiation (UV wavelength 365 nm) for 12 min to achieve a complete crosslinking of the PEGDA. After this process, the obtained hydrogels were transferred into the CaCl_2_ solution for the crosslinking of alginate for 2.5 h. The final sample obtained with this methodology has been called the solid hydrogel.

**Figure 9:**
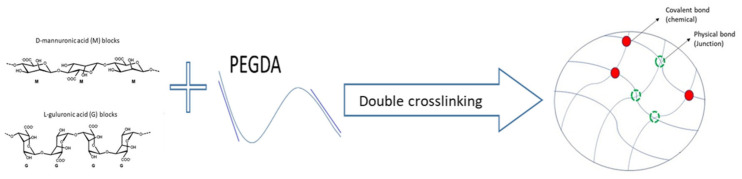
Schematic representation of the reaction leading to the double network crosslinking: alginate (**left**) reacting with polyethylene glycol diacrylate (**middle**) leading to polymer scaffold (**right**).

**Figure 10:**
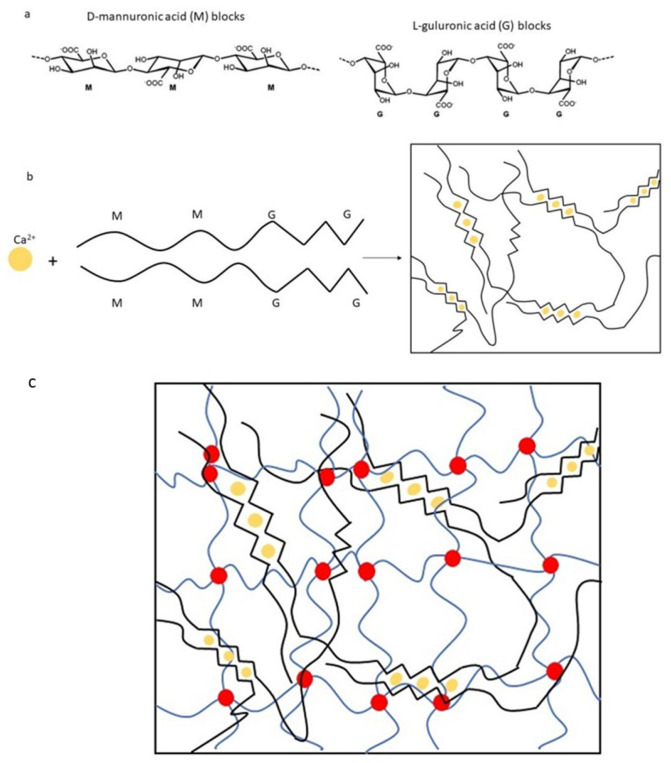
Schematic representations of the basic and crosslinked structures related to the alginate and double network hydrogels: (**a**) the alginate structure consisting of blocks of D-mannuronic acid (M) and L-guluronic acid (G) residues, (**b**) with ionic crosslinks, formed in contact with Ca^2+^, where the black line indicates the alginate chains and the yellow dots the points where the ionic cross-link occurs, (**c**) the double network structure, with the chemical conjunctions in yellow and the physical conjunctions in red.

Figure 9 shows the reaction of the double crosslinking of sodium alginate (SA) and polyethylene glycol diacrylate (PEGDA), resulting in the formation of a three-dimensional network structure of hydrophilic polymer chains. As shown in Figure 10a,b, the structure of alginate consists of linear copolymers containing blocks of D-mannuronic acid (M) and L-guluronic acid (G) residues [39]. These blocks can be composed of consecutive G residues (GGGGGG), consecutive M residues (MMMMMM), and alternating M and G residues (GMGMGM). When in contact with different divalent cations, such as calcium (Ca^2+^), the G blocks on different polymer chains form ionic crosslinks via the carboxyl groups (-COO^−^), resulting in the formation of a hydrogel matrix [40]. Due to its structure, which resembles that of extracellular matrices found in living tissues, alginate hydrogels are commonly utilized in tissue engineering applications [41]. Figure 10c provides a schematic representation of the double network structure produced by the photopolymerization of PEGDA (chemical crosslinking) and the ionic crosslinking of alginate (physical crosslinking) with calcium ions.

#### 3.1.4. Porous Hydrogel Preparations

**Figure 11:**
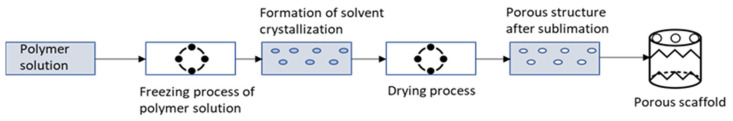
Illustration of the freeze-drying process.

After the hydrogel preparation process, the resulting gels were transferred to deionized water to rinse any residual components and then kept in water overnight to stay in wet conditions and to let them swell until equilibrium. The next day, the sample was frozen (−80 °C) and put in the freeze dryer overnight (for 18 h). Then, the sample was kept at a pressure (p) and temperature (T) below the triple point of water. By keeping the pressure below the triple point value and allowing the temperature to increase, the ice sublimated, which caused the formation of pores and, finally, the porous hydrogel. Figure 11 shows the freeze-drying process. The sample obtained via the freeze-drying technology in this work has been identified as the porous hydrogel. 

#### 3.1.5. Cell Culture

The nHDF fibroblast cell line is maintained in culture with complete medium (90% (*v*/*v*) and 10% (*v*/*v*) of fetal bovine serum). In order to promote cell proliferation, fibroblasts were subjected to incubation with conditions set at a controlled environment, with the temperature maintained at 37 °C in an atmosphere containing 5% CO_2_.

**Figure 12:**
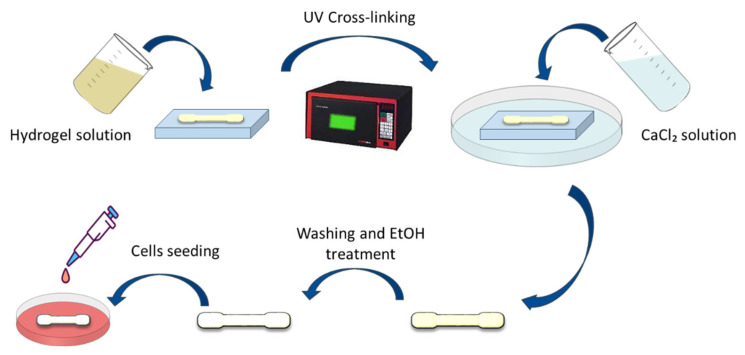
Scheme of the sample production process and cell seeding.

In preparation for cellular seeding, all material samples, whether in a solid or porous hydrogel format, underwent ethanol treatment. Various sterilization methods, including autoclaving and UV irradiation, were evaluated. However, only the treatment with 70% ethanol yielded satisfactory results in terms of sterilization efficiency since it ensured that all microorganisms (such as bacteria, fungi, etc.) were eradicated. The sample was soaked in ethanol for 1 h, after which the ethanol was refreshed, followed by 3 sequences of 30 min of soaking and subsequent ethanol refreshment. Then, the ethanol was removed, and the sample was left to dry overnight. Subsequently, cells were seeded onto the material’s surface, and a 48 h incubation period ensued within the aforementioned controlled environment. It is pertinent to note that in the experimental conditions, a standardized seeding density of 50,000 cells was employed on each cm^2^ of the sample. Figure 12 shows all the steps for the sample preparation. In the porous hydrogel sample, the freeze-drying procedure is between the CaCl_2_ solution and the washing process. 

### 3.2. Experimental Methodology

The term “cell-viable” pertains to hydrogel scaffolds capable of supporting cell viability throughout the tissue engineering process. Before testing the biocompatibility of the sample, it is necessary to verify that the mechanical integrity of the produced material fits the tissue engineering requirements. In addition, the following section describes the experimental setup used for the ground tests and the parabolic flights, as well as the different tests that were carried out to evaluate the properties of the samples.

#### 3.2.1. Experimental Setup

**Figure 13:**
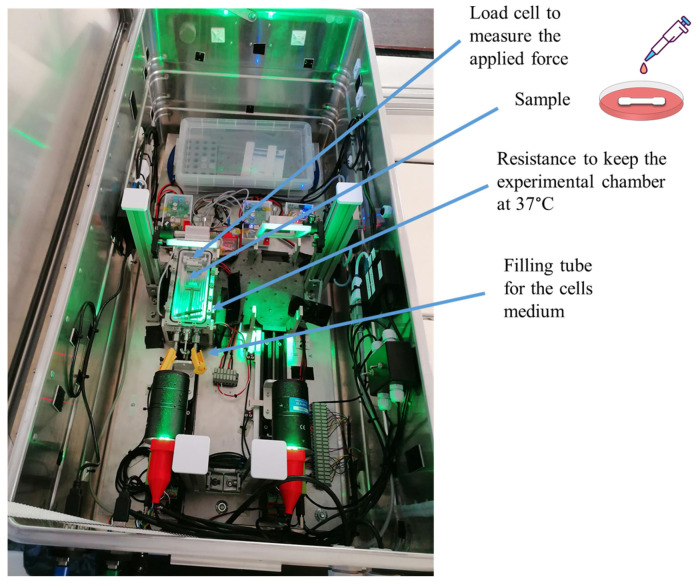
Experimental setup used for the 83rd ESA Parabolic Flight Campaign developed for the “HydroG” experiment, a name given by ESA to the experiment on board of the AirZeroG Plane. The same test rig was used for the ground experiments.

The experimental setup was designed and manufactured in the CREST (Centre for Research and Engineering in Space Technologies) laboratories and is shown in Figure 13. It comprises:

- A load cell (from 0 to 100 g) to measure the applied force generated during the tensile stress test;
- A motor to generate the tensile stress;
- An experimental chamber where the sample is to be inserted;
- A heating resistance to keep the experimental chamber at 37 °C;
- A tube to fill the chamber with the cell medium.

**Figure 14:**
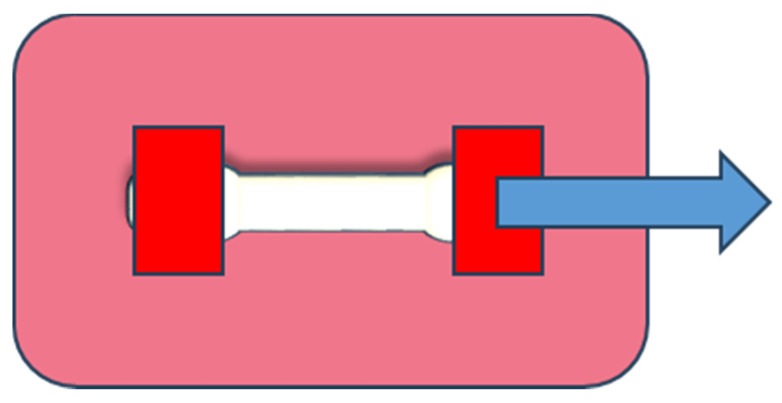
Force direction during the tensile stress experiment.

The sample in the experimental chamber is fixed between two grips, and the pulling force is applied from one side, as shown in Figure 14.

All the tests were performed on the ground and during the parabolic flight campaign as a part of the 83rd European Space Agency (ESA) Parabolic flight campaign (Bordeaux-Mérignac Airport, France) on-board the Airbus A310 Zero Gravity (ZeroG) aircraft.

#### 3.2.2. Experimental Procedure

The experiments were performed in both normal gravity (on-ground using the same test rig) and in altered gravity. For the ground test, the same set-up was used in the CREST laboratories as in the parabolic flights to have the same conditions except for the gravity level. The experiments were also performed in both relaxed and stressed conditions. The relaxed condition corresponds to applying no tensile force to the samples during the experiments, while for the stressed condition, the elongation of the sample was fixed at 2 mm, which corresponds to a force of 0.5 N. Finally, both the solid hydrogel and porous hydrogel configurations were used. The standard test, dubbed here as solid hydrogel, was conducted to study the influence of altered-gravity and mechanical stress conditions on the fibroblast morphology adhering to a biomimetic material. The 3D tests describe the study of the influence of altered-gravity and mechanical stress conditions on the morphology of fibroblast cells that are seeded within the porous structure of a biomimetic hydrogel. Finally, note that the variation in gravity can also be described as a type of stress to the fibroblast. So, for each solid hydrogel and porous hydrogel configuration, we would have different levels of mechanical and gravity-related stresses.

#### 3.2.3. Cell Viability and Morphology

For the cells’ viability, Thermo Fisher’s “LIVE/DEAD TM Cell Imaging Kit” in conjunction with the “Nikon microscope’s Epi Fluorescence attachment” was used. The fluorescence kit contains calcein, AM, a cell-permeant dye (to indicate live cells), and BOBO-3 Iodide (to indicate dead cells). In this work, we took advantage of the ability of the cells to appear red when dead and green when living. The colors appear as a consequence of a reaction with the aforementioned dye solutions that have been poured on the cells. The cells are then stored for 30 min in the incubator and then observed under a fluorescence microscope.

### 3.3. Mechanical Properties

To assess whether the mechanical properties of the developed material met the requirements of tissue engineering applications, tensile tests were conducted. To study the mechanical characteristics of the specimen, we conducted tensile tests utilizing the Uniaxial SHIMADZU AUTOGRAPH AGS-X tensile machine, operating at a crosshead speed of 0.02 mm/s. This machine recorded both the applied force and the resulting displacement, from which load data were obtained and subsequently converted into stress and strain values based on the sample dimensions. The stress (σ) was determined using the equation $\sigma =\frac{F}{A}$, where F represents the force applied and A denotes the cross-sectional area of the sample, calculated as the product of its thickness and width. The strain (ε) was calculated using the equation $\varepsilon =\frac{∆l}{l_{0}}*100$, where ∆l represents the change in length from the initial ($l_{0}$) to the final state of the sample. To determine the material’s elasticity, specifically the Young’s modulus (YM), we analyzed the initial linear segment of the stress–strain curve, specifically between strains of 0.05% and 1.5%. These tensile tests were conducted at ambient temperature using dumbbell-shaped specimens. The selection of this shape aligns with the ASTM D412 standard [42], which was adapted to suit our experimental setup. It is worth noting that the dumbbell shape was chosen as it serves as a reliable indicator of the mechanical robustness of the samples.

### 3.4. Scanning Electron Microscopy for Hydrogel Morphology

The porous hydrogel morphology was characterized using SEM (Scanning electron microscope). The Hitachi SU-70, SEM-Feg (USA), was used to evaluate the sample’s morphology and pore diameter. Carbon was applied to the 2 mm thick, 1 cm^2^ samples to perform the SEM analysis. Pictures were taken with a 5 kV accelerating voltage at working distances varying from 4.0 to 6.0 mm.

## 4. Conclusions and Future Perspective

This study contributes valuable insights into the interplay between microgravity, mechanical stress, and cellular morphology, with implications for understanding cellular behavior in space-related environments and tissue engineering applications. There are tests in the literature that present the microgravity effect on human cells [43]. Despite the emerging interest in the impacts of space travel on human health [7,8] and, by extension, its impact on the biology of living organisms, our comprehension of its implications for tissue engineering remains somewhat limited. The field of tissue engineering in the context of space exploration is nascent yet promising, characterized by an array of ongoing research initiatives [44,45]. The unique microgravity environment offered by space holds the promise of reduced mechanical loads on tissues, altered fluid dynamics, and enhanced potential for cell growth, which offers an array of advantages for tissue engineering and regenerative medicine. However, pursuing these opportunities entails overcoming several formidable obstacles, including the fiscal and logistical challenges associated with conducting space research, ensuring astronauts’ safety, and preserving engineered tissues’ integrity during transit to and from orbit [46]. 

While the parabolic flight provides a first look at how modified gravity affects the morphology of cells attached to a biocompatible material under mechanical stress, more tests under extended, modified gravity conditions—like those found in IceCube (a platform that allows performing experiments in space, confined to a cube for space limitations) or on board the International Space Station (ISS)—are necessary.

## Figures and Tables

**Table 1:** Summary of the results.

Situation	Result
Optimal: Relaxed on Ground	Elongated cells in star shape
Single stress: Tensile elongation	The cell’s morphology results in multicellular spheroids, and the cells start moving towards the sample edge
Single stress: Altered Gravity	The cell’s morphology results in multicellular spheroids, and the cells start moving towards the sample edge
Double stress: Tensile elongation + Altered Gravity	The cell’s morphology results in multicellular spheroids, and the cells start moving towards the sample edge. Compared to the single stress situation, this phenomenon is even stronger

## Data Availability

Data are available upon reasonable request from the authors.

## Abbreviations

TE	Tissue Engineering
TERM	Tissue engineering and regenerative medicine
SA	Sodium alginate
PEGDA	Poly(ethylene glycol)-diacrylate
I2959	Igracure 2959 (2-Hydroxy-4’-(2-hydroxyethoxy)-2-methylpropiophenone)
CaCl_2_	Calcium chloride dehydrate (CaCl_2_∙2H_2_O)
Gel	Gelatine
ISS	International Space Station
nHDF	Normal Human Dermal Fibroblasts
FBS	Fetal Bovine Serum
PBS	Phosphate-buffered saline
ECM	Extracellular Matrix
